# A Randomized Study of Food Pictures-Influenced Decision-Making Under Ambiguity in Individuals With Morbid Obesity

**DOI:** 10.3389/fpsyt.2020.00822

**Published:** 2020-09-11

**Authors:** Marek Lescher, Elisa Wegmann, Silke M. Müller, Nora M. Laskowski, Ruth Wunder, Susana Jiménez-Murcia, Gregor R. Szycik, Martina de Zwaan, Astrid Müller

**Affiliations:** ^1^Department of Psychosomatic Medicine and Psychotherapy, Hannover Medical School, Hannover, Germany; ^2^General Psychology: Cognition and Center for Behavioral Addiction Research (CeBAR), University of Duisburg-Essen, Duisburg, Germany; ^3^Surgical Department, Clementinenhaus, Hannover, Germany; ^4^Department of Psychiatry, University Hospital Bellvitge-IDIBELL and CIBEROBN, Barcelona, Spain; ^5^Department of Psychiatry, Social Psychiatry and Psychotherapy, Hannover Medical School, Hannover, Germany

**Keywords:** obesity, craving, cue-reactivity, food addiction, decision-making, Iowa Gambling task

## Abstract

**Background and Aims:**

In addition to craving responses to salient food cues, the anticipation of short-term rewarding consumption of palatable food may overrun the anticipation of long-term negative consequences of obesity. The present investigation addressed the potential interplay of food cravings and decision-making abilities in individuals with obesity.

**Method:**

Study 1 included 107 bariatric surgery candidates with class 2/3 obesity (OB-group) and study 2 included 54 individuals with normal weight/pre-obesity (nonOB-group). In both studies, standardized questionnaires concerning food cravings, food addiction, and psychopathology were administered. A cue-reactivity paradigm was used to measure craving responses toward semi-individualized images of highly palatable, processed food/fruit (appetitive food cues) compared to images of raw vegetables (non-appetitive food cues). Decision-making was measured with a modified computerized version of the Iowa Gambling Task (IGT) with food pictures. Both groups were divided into two subgroups that were randomized to different IGT conditions. In one IGT condition the advantageous IGT card decks were covered by pictures of palatable, processed food or fruit and the disadvantageous decks by images of raw vegetables (= congruent condition), and in the other IGT condition *vice versa*.

**Results:**

Participants in the OB-group admitted on average higher craving responses toward palatable, processed food or fruit cues compared to pictures of raw vegetables. This was not the case in the nonOB-group. Contrary to our hypothesis, decision-making performance in both groups was worse when pictures of palatable, processed food or fruit were associated with advantageous IGT card decks compared to performance when those pictures were linked to the disadvantageous decks. The interference effect of food pictures processing on advantageous decision-making has been observed particularly in those individuals of the OB-group who exhibited high craving responses toward palatable, processed food cues or high levels of food addiction.

**Discussion:**

The results indicate that food pictures processing interferes with decision-making, regardless of weight status. Opposed to the hypothesis, stronger tendencies to avoid than to approach pictures presenting processed, tasty food were observed. Further research should examine how cognitive avoidance tendencies toward processed, high energy food and approach tendencies toward healthy food can be transferred to real life situations.

## Introduction

Over the last decades, obesity has become a serious public health challenge (1, 2). In 2016, the worldwide prevalence of obesity was about 13% which is almost three times as much as in 1975 (2). Eighteen percent of adults in Germany are obese (3). The national rates of obesity in some US states are approaching almost 40% (4). This is worrisome given that obesity increases the risk for weight-related somatic disorders (e.g., type 2 diabetes, metabolic syndrome, cardiovascular diseases), mental health disorders (e.g., depression), and mortality (5–7). On the level of the individual’s metabolism, overweight and obesity occur when calorie intake exceeds calorie expenditure (8). Therefore, conventional treatments of obesity involve decreasing energy intake (diet), increasing energy expenditure (physical activity, exercising), and offering structured behavioral-change programs.

The cause of obesity is multifaceted (9). Over the last decade, research has stressed the importance of addiction-like responses to highly palatable, processed food in the development and maintenance of unhealthy eating habits (10–13). After repeated exposure, cues that are related to tasty food (e.g., sight or smell of food) may become conditioned attractive and “wanted” stimuli for food consumption, evoking an intense desire to eat (14–17). Thus, food cue-induced craving can be defined as an inclination to approach palatable food, resulting in sustained overconsumption (18, 19). In addition to craving responses toward salient food cues, the anticipation of short-term rewarding eating of delicious food may overrun the anticipation of long-term adverse consequences of overweight and obesity such as weight gain, somatic comorbidities, etc. Making disadvantageous choices may contribute to overeating and obesity (20–27). It has been argued that individuals with obesity typically approach appetitive food stimuli (i.e. images of palatable, processed food) more than healthy food stimuli (i.e. images of raw vegetables) as a function of their addictive eating habits (28). Experimental studies concerning approach tendencies toward pleasant food stimuli in individuals with obesity, however, revealed mixed results (28, 29). One reason for the inconsistencies may be that past studies did not individualize the food stimuli with respect to the person’s food preference.

The present study addressed the potential interplay of food cravings and decision-making abilities in patients with obesity. It investigated whether the exposure to pictures of palatable, processed food *vs.* healthy food interferes with decision-making in the Iowa Gambling Task (IGT) (30). The IGT has been widely used to investigate real-world decision-making under uncertainty (i.e. the probability of the occurrence of an outcome is unknown) and to investigate the preference for short-term rewarding choices in a laboratory setting in several clinical populations (30), including individuals with obesity (20, 21) and bariatric surgery candidates (22, 27, 31, 32). In this behavioral task, participants are exposed to four virtual card decks that are associated with theoretical financial gains, and occasionally with financial losses. The participants are given a fictitious starting capital. Without being informed which decks are more valuable, they are instructed to pick cards with the aim to increase the financial profit until they are told to stop. Two card decks offer high financial gains, but also high losses that exceed the gains in the long run, and the other two decks yield moderate gains and losses that in total do not exceed the gains. Sustained choosing cards from the first two decks is considered “disadvantageous” decision-making, while choosing cards from the latter two decks is linked to “advantageous” decisions.

Although most IGT studies indicated altered decision-making in individuals with obesity (25), it remains unclear whether food preferences interfere with general decision-making. The present study attempted to approximate natural food-associated decision-making situations of individuals with obesity by using a modified IGT version where card decks were covered with semi-individualized food pictures, taking into account participants’ preferences for specific palatable food. Modified versions of the IGT with addiction-related (i.e. pornography- or shopping-related) pictures either on the advantageous decks or on the disadvantageous decks and neutral control pictures from the International Affective Picture System (IAPS) (33) on the opposing ones have already been utilized by Laier et al. (34) and Trotzke et al. (35). Participants who played the IGT with the addiction-related pictures displayed on the advantageous decks performed better than the other group with the addiction-related pictures on the disadvantageous decks (34, 35). For the present study, in the congruent condition, the advantageous IGT decks were covered by images of palatable, processed food or fruit, and the disadvantageous decks by pictures of raw vegetables. In the incongruent condition, the pictures of palatable, processed food or fruits were displayed on the disadvantageous card decks and the vegetable pictures on the advantageous decks. Food pictures were semi-individualized in accordance with the participants’ preferences for specific palatable food. Non-appetitive food cues instead of neutral IAPS pictures were used as control cues because the present study focused on participants’ responses toward pictures of palatable, processed food *vs.* healthy food (and not on food cues *vs.* non-food cues).

The empirical work presented here consists of two experimental studies. Study 1 included individuals with class 2 or 3 obesity. Against the background of the findings of study 1, we performed a subsequent study 2, sampling individuals with normal-weight or pre-obesity.

## Study 1

Study 1 proposed and tested the idea that in individuals with class 2 or 3 obesity the presentation of food pictures will interfere with their decision-making performance. More specifically, the following hypotheses were raised:

1. Participants rate pictures of palatable, processed food or fruit as more appetitive than those showing raw vegetables.
2. The decision-making performance in the IGT differs between the congruent and the incongruent IGT conditions. Participants in the congruent IGT condition (i.e. pictures of palatable, processed food or fruit on advantageous decks) show more advantageous decision-making than participants in the incongruent IGT condition (i.e. pictures of palatable, processed food or fruit on disadvantageous decks).
3. Between-group differences in the IGT performance (congruent *vs.* incongruent) are moderated by craving responses/food addiction symptoms. Higher craving responses towards palatable, processed food or fruit/more food addiction symptoms are related to more disadvantageous decision making especially in the incongruent IGT condition, because of more frequent choices of disadvantageous decks (containing pictures of palatable, processed food or fruit).

### Materials and Method

#### Participants With Obesity

The study protocol met ethical and legal aspects of research involving human subjects in accordance with the Declaration of Helsinki (Institutional Review Board approval No: 7556, Hannover Medical School, Germany). The study was registered in the German Clinical Trials Register (DRKS00012658).

Participants were recruited within preoperative bariatric surgery evaluations at two hospitals (both located in Hannover, Germany). Inclusion criteria were class 2 or 3 obesity (i.e. BMI ≥ 35 kg/m^2^), age 18 years or older, and sufficient German language skills. Exclusion criteria were impairments in cognitive functions as measured with the Modified Card Sorting Task (MCST; see below), psychosis, current substance dependence (except tobacco), acute suicidal ideations, sensory impairment, and scoring equal or above 50 on the numeric hunger scale (see below). *A priori* power analysis assuming a medium effect size for between-group differences based on the findings of Laier et al. (34) indicated that a total sample size of 120 participants is sufficient to reach 80% power when employing the .05 criterion of statistical significance.

Data were collected between October 2017 and April 2018. Participants were assured that the assessment would be completely independent from any preoperative evaluation or care, assessors would not be included in the routine preoperative evaluation or care, and that information provided for the present study would not be forwarded to the interdisciplinary bariatric surgery team.

The initial sample included 125 bariatric surgery candidates who were randomly assigned to the congruent (*n* = 62) or incongruent (*n* = 63) IGT condition. Data from 18 participants were not included into the analyses due to the following reasons: BMI < 35 kg/m^2^ (*n* = 1), test instruction was not understood (*n* = 1), scoring on the numeric hunger scale > 50 (*n* = 6), more than 20 perseverative errors in the MCST (*n* = 2), and technical problems with the IGT (*n* = 2). Six participants were excluded because they had constantly picked cards from a certain deck that resulted in one empty IGT card deck after 60 out of 100 trials (*n* = 3 in each IGT condition). The final sample consisted of 107 participants; of those 52 individuals were assigned to the congruent and 55 to the incongruent IGT condition.

#### Study Procedure

**Figure 1:**
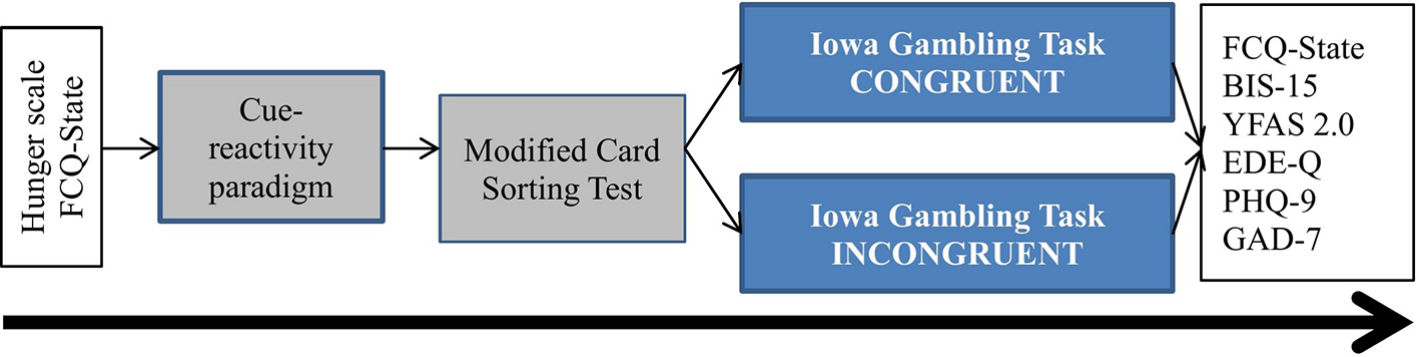
Study procedure for study 1 and study 2. FCQ, Food Craving Questionnaire; BIS-15, Barratt Impulsiveness Scale; YFAS, Yale Food Addiction Scale; EDE-Q, Eating Disorder Examination-Questionnaire; PHQ-9, 9-item depression Patient Health Questionnaire; GAD-7, 7-item Generalized Anxiety Disorder Scale.

**Figure 1** demonstrates the one-session laboratory procedure that lasted between 60 and 90 min. The assessments were conducted by two independent assessors (authors ML and NL) who were not included in the preoperative evaluation or care. All participants were asked to come to the experimental session in a saturated state. After given written informed consent, participants provided demographic information, rated their hunger on a numeric rating scale, and answered the Food Cravings Questionnaire-State (FCQ-State, see below). Then they were asked to choose one out of seven food categories of palatable, processed food or fruit in order to semi-individualize the visual food cues for the IGT with respect to the person’s food preference. After that, a cue-reactivity paradigm with the chosen pictures of palatable, processed food or fruit *vs.* pictures of raw vegetables was conducted (see below *Cue-reactivity paradigm*), and the MCST (see below) to assess executive functioning was carried out. Participants were then randomly assigned to one of two groups. One group played the IGT in the congruent condition and the other group played the IGT in the incongruent condition. After completing the IGT, all participants were asked to answer the FCQ-State for a second time and, additionally, they completed questionnaires assessing impulsivity and symptoms of food addiction as well as eating, depressive, and anxiety disorders. Finally, all participants were debriefed. All participants received a compensation of 50€.

#### Cue-Reactivity Paradigm

Visual food cues were taken from the “Food Pics” image database for experimental research on eating and appetite (36). **Supplementary Table S1** provides a list of food pictures used in the present study. All participants were asked to choose one out of the following seven food categories: cold cuts/cheese, pastries, hearty warm dishes, fast food, salty/nutty nibbles, sweets, fruit *via* Presentation software (Version 20.0, Neurobehavioral Systems Inc., Berkley, CA, USA). Each category consisted of 20 images. Pictures of the selected food category together with 20 images of raw vegetables were then presented in a randomized manner. Participants had to rate all pictures on valence (“How appetizing is this food to you in general?”, 1 = “not appetizing” to 5 = “very appetizing”) and urge to eat (“How strong is your desire to eat this food right now?”, 1 = “no urge” to 5 = “high urge”).

#### Modified Card Sorting Task (MCST)

The computerized version of the MCST was used to assess executive functions such as cognitive flexibility, feedback processing, and rule detection (37, 38). Dependent variables were the total number of non-perseverative errors and the total number of perseverative errors indicating deficits in feedback processing and categorizing abilities. Data from participants with more than 20 perseverative errors were dropped from analyses (see above).

#### Modified Iowa Gambling Task (IGT)

As mentioned above, a modified computerized version of the IGT was used with card decks covered either with the preferred pictures of palatable, processed food or fruit (also used in the cue-reactivity paradigm) or with images of raw vegetables. Using a between-group design, the total sample was randomly divided into two groups. One group played the IGT in the congruent condition, in which pictures of palatable, processed food or fruit were displayed on advantageous decks (decks C and D) and pictures of raw vegetables on the disadvantageous decks (decks A and B). The other group played the IGT in the incongruent condition with pictures of palatable, processed food or fruit being displayed on disadvantageous decks and pictures of raw vegetables displayed on advantageous decks. Participants completed a total of 100 trials (5 blocks of 20 trials). The main dependent variable was the overall IGT net score that is derived from the total number of cards chosen from the advantageous decks minus those chosen from the disadvantageous decks ([C + D] − [A + B]) (30). A positive net score indicates advantageous decision-making and a negative net score indicates disadvantageous decision-making. In addition to the overall IGT net score, separate net scores across the five IGT blocks were calculated to examine whether participants gradually learned which card decks are more valuable and whether they shifted their choices toward the advantageous decks over the course of the task.

### Questionnaires

#### Numeric Hunger Rating Scale

Participants were asked to rate their momentary feeling of hunger on a numeric rating scale that ranged from 0 (= “not hungry at all”) to 100 (= “very hungry”). This scale was displayed before the cue-reactivity paradigm. Participants with a hunger rating of >50 were excluded.

#### Food Cravings Questionnaire-State (FCQ-State)

The short German version of the FCQ-State was used to measure food cravings at state-level (39). The instrument consists of 15 items to be scored on a five-point scale ranging from 1 (= “strongly disagree”) to 5 (= “strongly agree”). The FCQ-State was administered twice, at baseline and after completing the IGT. Internal consistency was excellent with Cronbach’s α being .96 at baseline and .97 following the IGT.

#### Yale Food Addiction Scale 2.0 (YFAS 2.0)

The German translation of the YFAS 2.0 (40) was used to assess addiction-like eating. The YFAS 2.0 is comprised of 35 items that measure symptoms of food addiction in accordance with the DSM-5 criteria for substance use disorders (41), adopted for highly palatable food (e.g. chocolate, rolls, pasta, chips, hamburgers, etc.). A continuous count score that reflects the number of fulfilled food addiction criteria can be calculated ranging from 0 to 11 (Kuder-Richardson α for the dichotomous scores of the 11 food addiction symptoms .84). The following severity levels of food addiction can be differentiated based on the symptom count: mild (i.e. 2–3 criteria), moderate (i.e. 4–5 criteria), and severe (i.e. 6–11 criteria) (40, 42). The three groups with mild, moderate, and severe food addiction symptoms were collapsed forming the group with an YFAS 2.0 diagnosis of food addiction (i.e. exhibiting at least two symptoms of food addiction plus significant impairment/distress).

To characterize the samples in more detail, additional questionnaires were administered. Impulsivity was measured using the German Barratt Impulsiveness Scale-short version (BIS-15; α = .81) (43). Eating disorder symptoms were measured with the German version of the Eating Disorder Examination-Questionnaire (EDE-Q; α = .86; cutoff for being at-risk for an eating disorder ≥2.3) (44). The German versions of the nine-item depression Patient Health Questionnaire (PHQ-9; α = .85; cutoff for major depressive disorder ≥10) (45) and the seven-item Generalized Anxiety Disorder Scale (GAD-7; α = .90; cutoff for anxiety disorder ≥10) (46) were used to assess symptoms of depressive or anxiety disorders.

### Statistical Analysis

Statistical analyses were carried out with IBM SPSS Statistics Version 24.0 (IBM Corp., Armonk, NY, USA). Participants allocated to the congruent IGT condition were compared with participants assigned to the incongruent IGT condition with respect to demographics, appetitive food pictures selection/ratings, questionnaire scores, and MCST performance using independent *t-*tests or χ^2^-tests, as appropriate. To test the first hypothesis, within-group differences in the ratings of appetitive food pictures and pictures of raw vegetables were analyzed using paired-samples *t*–tests. In addition, multivariate analysis of variance (MANOVA) with the four pictures ratings as dependent variables (valence/urge toward palatable, processed food or fruit/raw vegetables) and IGT-condition (congruent *vs.* incongruent) as between-factor were conducted. Between-group comparisons (congruent *vs.* incongruent) of questionnaire scores, IGT net scores (second hypothesis), and other variables were performed using analysis of variance (ANOVA). Repeated measures (“IGT block” 1 to 5) ANOVA with the between subjects factor “group” (congruent *vs.* incongruent) were used to test whether the task performance across the five blocks differed between the two IGT conditions. With respect to the third hypothesis, moderated regressions and simple slope analyses were performed to check potential two-way interactions of IGT condition and food craving responses/food addiction symptoms on decision-making (i.e. overall IGT net score). For the regression analyses, the interaction terms were calculated by using the centered variables of the predictor and the moderator variable (47). With respect to the simple slope analyses, the respective predictor/moderator variables were grouped one *SD* above or below the mean to explore the slopes of the regression lines representing high or low craving/food addiction symptoms.

The significance level was set to *p* <.05. All tests were two-tailed. Cohen’s *d* (*t-*test), φ coefficients (χ^2^-test) and partial η^2^ (ANOVA) are reported as effect sizes, while values of *d* > 0.2, φ > 0.1, and η^2^ > 0.01 are considered as small effects, *d* > 0.5, φ > 0.3, and η^2^ > 0.06 a moderate effects, and *d* > 0.8, φ > 0.5, and η^2^ > 0.14 as large effects (47).

### Study 1: Results

#### Demographic and Clinical Variables

The sample consisted of 107 bariatric surgery candidates (women *n* = 77, 72%) with a mean age of 41.36 years (SD = 11.65, *range* 18–67, *Mdn* = 42.00). The majority had class 3 obesity (*n* = 92, 86%), the remaining participants suffered from class 2 obesity (*n* = 15, 14%). The following prevalence estimates were found based on questionnaires’ cutoffs: food addiction 48.6% (*n* = 52), eating disorder 72.9% (*n* = 78), major depressive disorder 42.1% (*n* = 45), and anxiety disorder 22.4% (*n* = 24). **Supplementary Table S2** displays the comparisons between participants in the congruent compared to those in the incongruent IGT condition with respect to demographic and clinical variables. No significant between-group differences were found with regard to age, gender, BMI, level of impulsivity, as well as regarding the symptom severity of food addiction, eating, depressive or anxiety disorders.

#### Subjective Food Pictures Ratings and Food Cravings

**Supplementary Figure S1** shows the distribution of selected food categories of palatable, processed food or fruit for both IGT conditions. Individuals in the congruent IGT condition did not differ significantly from those in the incongruent condition with regard to preferred food, whereas the φ coefficient indicated an almost medium effect (χ^2^ = 8.76, df = 5, *p* = .119, φ = .29). It is noteworthy that none of the participants had selected the food category “nibbles.” Paired-samples *t*-tests suggest that participants in both groups exhibited the pictures of the selected processed food or fruit as more positive than images of raw vegetables (total sample: *t* = 6.70, *df* = 106, *p* <.001, *d* = 0.88; congruent: *t* = 5.80, *df* = 51, *p* <.001, *d* = 1.10; incongruent: *t* = 3.96, *df* = 54, *p* <.001, *d* = 0.73). They further admitted a higher “urge to eat” toward those pictures compared to images of raw vegetables (total sample: *t* = 5.41, *df* = 106, *p* <.001, *d* = 0.50; congruent: *t* = 5.92, *df* = 51, *p* <.001, *d* = 0.56; incongruent: *t* = 2.61, *df* = 54, *p* <.001, *d* = 0.39). MANOVA with the four pictures ratings as dependent variables (valence/urge; palatable, processed food or fruit/raw vegetables) showed no main effect of group (congruent *vs.* incongruent) (Wilk’s λ *F*(4,102) = 0.97, *p* = .498, η² = .03).

No significant between-group differences (congruent. *vs.* incongruent.) were observed with regard to the level of hunger, food pictures ratings and food cravings (see **Table 1**). Within-group comparisons indicated higher food cravings as measured with the FCQ-State following the experimental procedure compared to baseline in individuals in the congruent IGT condition (*t* = 2.18, df = 50, *p* = .034, *d* = 0.15). No change in FCQ-State scores was found in the incongruent condition (*t* = .77, df = 54, *p* = .444, *d* = 0.04). Repeated measures ANOVA with FCQ-State scores at baseline *vs.* following the experiment as within factor “time” and IGT “condition” (congruent *vs.* incongruent) as between factor did not suggest a significant “time-by-condition” effect (Wilk’s λ = 0.98, *F*(1, 104) = 1.58, *p* = .212, η^2^ = .01).

**Table 1:** Comparison of hunger, food cravings, food pictures ratings, and general cognitive functions for individuals with obesity playing the modified IGT in the congruent *vs.* the incongruent condition (study 1).

		IGT condition		*t*	*p*	*d*
	Total sample	Congruent	Incongruent			
	*N* = 107	*n* = 52	*n* = 55			
	*mean (SD)*	*mean (SD)*	*mean (SD)*			
Hunger^a^	6.49 (10.31)	6.69 (10.33)	6.29 (10.37)	.20	.842	.04
Appetitive food pictures						
Rating—valence	3.75 (0.69)	3.83 (0.56)	3.67 (0.79)	1.24	.216	.24
Rating—urge to eat	2.03 (1.02)	2.10 (1.01)	1.96 (1.03)	.67	.502	.13
Raw vegetables pictures						
Rating—valence	3.10 (0.78)	3.12 (0.72)	3.07 (0,84)	.33	.741	.06
Rating—urge to eat	1.57 (0.67)	1.54 (0.55)	1.61 (0.78)	.53	.595	.10
FCQ-State						
Baseline^b^	27.37 (12.16)	28.27 (11.68)	26.53 (12.64)	.74	.463	.14
Post	29.16 (13.97)	31.10 (14.11)	27.33 (13.71)	1.40	.164	.27
MCST						
Correct responses	29.37 (9.96)	29.33 (9.89)	29.42 (10.11)	.05	.962	.01
Normal errors	11.23 (10.12)	10.58 (9.07)	11.85 (11.97)	.65	.517	.13
Perseverative errors	5.70 (4.70)	6.46 (4.77)	4.98 (4.56)	1.64	.104	.32

^a^Numeric Hunger Scale, ^b^df = 104, for all other comparisons df = 105.

IGT, Iowa Gambling Task; FCQ, Food Cravings Questionnaire; MCST, Modified Card Sorting Test.

#### Behavioral Tasks Performance: MCST and IGT

No significant between-group differences emerged regarding executive functions as measured with the MCST (see **Table 1**). In terms of decision-making, participants in the congruent condition had a lower overall IGT net score (*mean* = −21.04, *SD* = 28.69) than participants in the incongruent condition (*mean* = 27.49, *SD* = 35.08). An independent *t*-test showed that the difference in overall IGT net score means was statistically significant (*t* = 7.81, df = 105, *p* <.001) with large magnitude (*mean difference* = −48.53, 95% CI: −60.85 to −36.20, *d* = 1.51).

**Figure 2:**
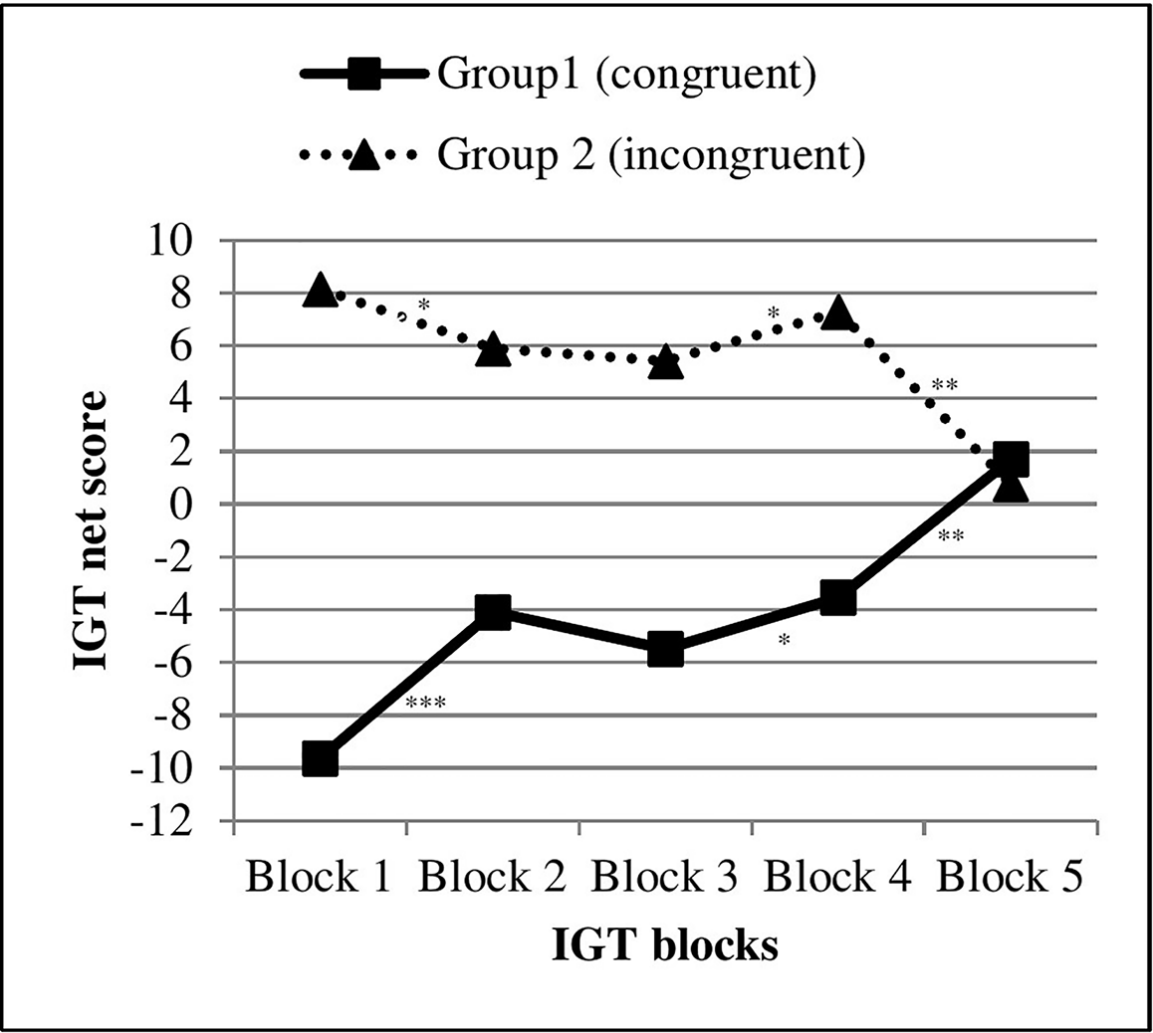
Task performance of individuals with obesity across five blocks in the congruent *vs.* incongruent IGT condition (study 1). IGT, Iowa Gambling Task, ***p < .001, **p < .01, *p < .05.

**Figure 2** displays the IGT net scores across the five IGT blocks for each condition. Separate repeated measures ANOVA for each IGT condition revealed significant main effects of block, indicating changes in IGT net scores across the five blocks in the congruent (*F*(4, 48) = 10.39, *p* <.001, η^2^ = .46) as well as in the incongruent (*F*(4, 51) = 4.75, *p* = .002, η^2^ = .27) condition. To compare the IGT learning curves between the two conditions, repeated measures ANOVA with total IGT net scores across the five blocks as within factor and IGT condition (congruent *vs.* incongruent) as between factor were performed. The result indicates a significant “block-by-condition” effect (Wilk’s λ = 0.68, *F*(4, 102) = 11.95, *p* <.001, η^2^ = .31). Descriptives and test statistics for the between-group comparisons (congruent *vs.* incongruent) are reported in **Supplementary Table S3** and for pairwise within–group comparisons in **Supplementary Table S4**.

#### Moderating Variables on Decision-Making Performance

Bivariate correlations of overall IGT net scores with hunger, food pictures ratings, food cravings, and clinical variables for each condition are shown in **Supplementary Table S5**. In the incongruent condition, negative correlations of the overall IGT net score with food cravings variables (i.e. “urge to eat” toward appetitive food pictures and FCQ-State baseline/post) and YFAS 2.0 food addiction symptoms were found. In the congruent IGT condition, only the EDE-Q mean total score was negatively related to the overall IGT net score. Notably is the difference in correlations of the IGT net score and FCQ-State post score between the congruent and incongruent IGT version (*r_congr_* = .16 *vs. r_incongr_* = −.51, *z* = 3.64, *p* = <.001).

**Figure 3:**
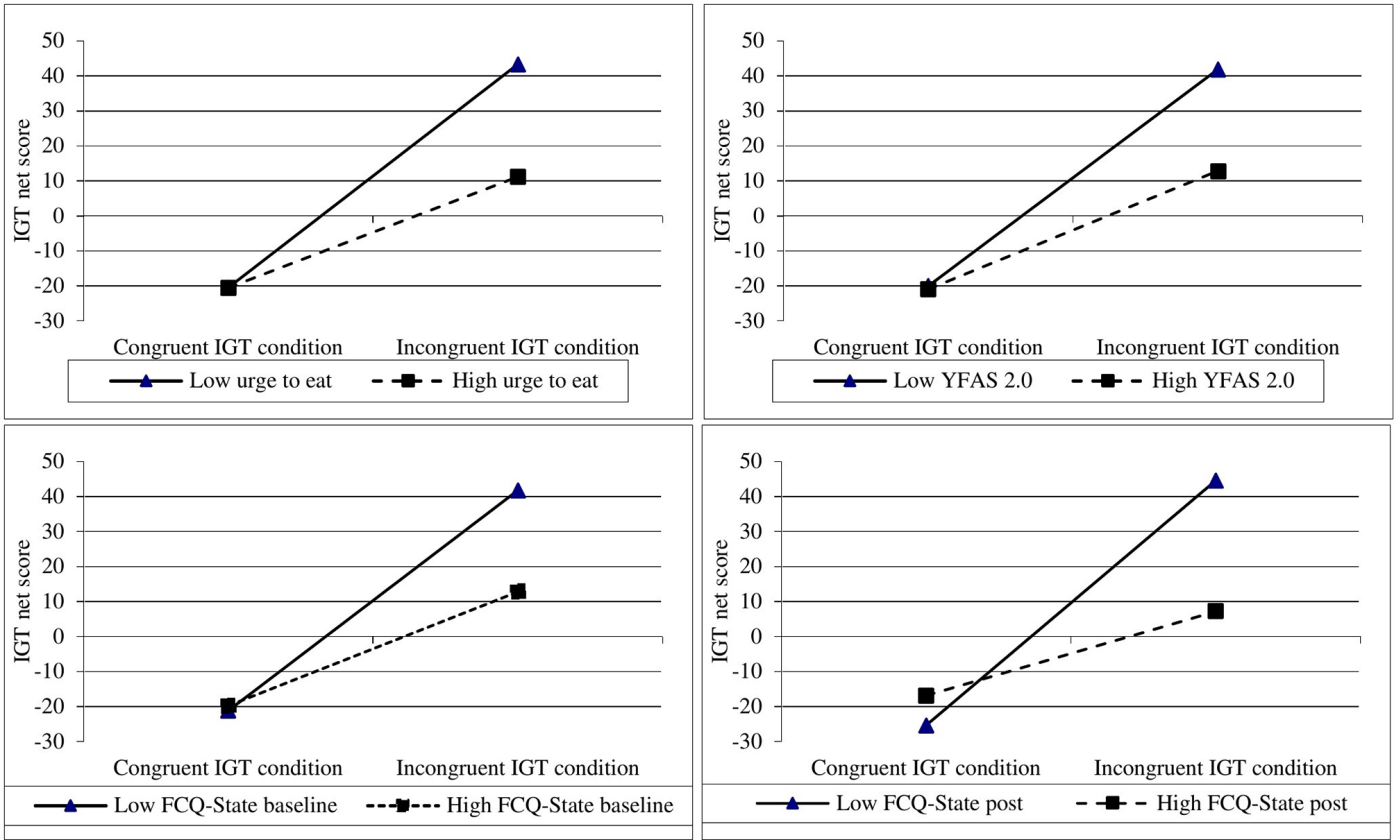
Graphical illustrations of simple slopes showing two-way interactions of IGT condition and food craving responses/food addiction symptoms on decision-making in patients with obesity (study 1). IGT, Iowa Gambling Task; YFAS, Yale Food Addiction Scale; FCQ, Food Craving Questionnaire.

To test the third hypothesis on the interaction effect between IGT condition and food cravings/food addiction symptoms in predicting the decision-making outcome, separate moderated regressions were conducted for the following potential moderators: “urge to eat” (from the food pictures rating), FCQ-State baseline, FCQ-State post, and symptoms of food addiction (YFAS 2.0). For all models, the overall IGT net score was the dependent variable. IGT condition was entered as first predictor, the potential moderator as second predictor, and the interaction term as third predictor. As can be seen in **Table 2**, significant interaction effects were found between experimental condition and all moderators in predicting the overall IGT net score. **Figure 3** illustrates the simple slopes that were significant from zero for all regression lines (*t* ≥ 2.93,.05 > *p* ≥.004).

**Table 2:** Summary of moderated regression analyses investigating the impact of craving responses, food addiction symptoms, or impulsivity on the relationship between IGT condition and decision-making outcome (dependent variable: overall IGT net score) in individuals with obesity (study 1).

Predictor/Moderator variables	*F*	*R^2^*	*B*	*SE*	β	*t*	*p*
Step 1	60.94^***^	.37					
Condition			48.53	6.22	.61	7.81	<.001
Step 2	35.98^***^	.41					
Condition			47.45	6.05	.59	7.84	<.001
Urge to eat			−8.06	2.97	−.20	−2.71	.008
Step 3	27.88^***^	.45					
Condition			47.54	5.87	.59	8.09	<.001
Urge to eat			−7.88	2.89	−.20	−2.73	.008
Condition × Urge to eat			−15.65	5.78	−.20	−2.70	.008
Step 1	59.62^***^	.36					
Condition			48.47	6.28	.60	7.72	<.001
Step 2	34.25^***^	.40					
Condition			47.38	6.15	.59	7.71	<.001
FCQ-State baseline			−.62	.25	−.19	−2.45	.016
Step 3	26.06^***^	.43					
Condition			47.55	6.00	.59	7.93	<.001
FCQ-State baseline			−.57	.25	−.17	−2.28	.025
Condition × FCQ-State baseline			−1.24	.50	−.19	−2.49	.014
Step 1	60.94^***^	.37					
Condition			48.53	6.22	.61	7.81	<.001
Step 2	34.10^***^	.40					
Condition			46.67	6.16	.58	7.58	<.001
FCQ-State post			−.49	.22	−.17	−2.23	.028
Step 3	31.10^***^	.47					
Condition			46.76	5.77	.58	8.10	<.001
FCQ-State post			−.52	.21	−.18	−2.49	.014
Condition × FCQ-State post			−1.63	.41	−.28	−3.94	<.001
Step 1	60.94^***^	.37					
Condition			48.53	6.22	.61	7.81	<.001
Step 2	34.43^***^	.40					
Condition			47.62	6.10	.59	7.80	<.001
YFAS 2.0			−2.25	.97	−.18	−2.32	.022
Step 3	25.74^***^	.43					
Condition			47.62	5.98	.59	7.97	<.001
YFAS 2.0			−2.37	.95	−.19	−2.49	.014
Condition × YFAS 2.0			−4.43	1.90	−.17	−2.33	.022
Step 1	60.94^***^	.37					
Condition			48.53	6.22	.61	7.81	<.001
Step 2	31.88^***^	.38					
Condition			50.19	6.28	.63	7.98	<.001
BIS-15			−.67	.45	−.11	−1.47	.145
Step 3	22.01^***^	.39					
Condition			49.95	6.26	.62	7.97	<.001
BIS-15			−.60	.46	−.10	−1.33	.188
Condition × BIS-15			−1.22	.91	−.10	−1.34	.185

IGT, Iowa Gambling Task; FCQ, Food Cravings Questionnaire State; YFAS 2.0, Yale Food Addiction Scale 2.0; BIS-15, Barratt Impulsiveness Scale, ^***^p < .001.

For means of comparison, additional regression models were calculated to investigate potential moderating effects of other variables (i.e. BIS-15, EDE-Q, PHQ-9, GAD-7). In none of the models significant interaction effects emerged. Exemplary, results for impulsivity (BIS-15) are reported in **Table 2**.

### Study 1: Discussion

The present sample of bariatric surgery candidates suffered from high levels of food addiction as well as eating, depressive and anxiety disorder symptoms that exceed questionnaire-based prevalence estimates in the general German population (44, 48, 49). The results are quite typical for patients who seek surgical treatment for obesity and consistent with past research (13, 27, 50, 51).

With respect to selected pictures of palatable, processed food, participants showed a preference for pictures presenting hearty food and sweets. In line with the first hypothesis, participants rated pictures of palatable, processed food or fruit as more appetitive than pictures showing raw vegetables. Based on past studies showing that participants who played the IGT with the appetitive visual cues on the advantageous decks (congruent condition) performed better than those who played the IGT with the appetitive cues on the disadvantageous decks (incongruent condition) (34, 35), we had hypothesized that participants in the congruent IGT version would perform better than participants in the incongruent condition. However, the result differed from our expectation. Individuals in the incongruent IGT condition, rather than those in the congruent condition, showed more advantageous decision-making, which has to do with their choices for those card decks linked to raw vegetables and the avoidance of decks with pictures of palatable, processed food or fruit. This result is, however, in line with a recent study in individuals with a substance use disorder, particularly patients with heroin addiction receiving opioid maintenance treatment compared to early abstinent users that was published after the completion of the present study (52). In that study, participants also seemed to avoid card choices associated with drug-related pictures and to prefer choices related to neutral IAPS images (52). This raises the question of whether images of palatable, processed food or drugs stimulate different responses while playing the IGT than pornography- or buying-shopping related pictures. Differences in outcome could further be explained by the fact that the studies of Laier et al. (34) and Trotzke et al. (35) included non-clinical samples, whereas the study of Kriegler et al. (52) examined patients receiving opioid maintenance treatment. The present sample consisted of bariatric surgery candidates who probably have been trained within preoperative counseling or previous conservative obesity treatments to prefer healthy food and avoid palatable, processed food. They may have tended towards socially desirable decisions by choosing the decks with healthy food. Given that they were seeking bariatric surgery, some of them may have thought that they were evaluated in order to determine their motivation to change. Those patients may have preferred card choices associated with healthy food pictures to enhance their chances of receiving a recommendation for surgery. However, all patients were assured that the assessors were not included in the bariatric surgery team and would not forward any information to the surgeons or mental health professionals who were in charge for the preoperative evaluation.

It is worth taking a closer look at the IGT learning curves. As illustrated in **Figure 2**, participants in both IGT conditions started with a clear preference for those cards with images of less appetitive raw vegetables on the backsides. Individuals in the congruent IGT version started with a low IGT net score because they preferred those choice options linked to raw vegetables. Participants in the incongruent condition started with a high IGT net score because they also preferred the cards with pictures of raw vegetables. The initial choices for healthy food pictures regardless of IGT condition may have been controlled by positive attitudes toward healthy food (i.e. raw vegetables) and/or prejudices against palatable, processed food. Of particular interest is that participants in both conditions then gradually shifted their choices to card decks covered by appetitive food pictures. Although IGT net scores across the five blocks changed in both conditions, only the group playing the congruent IGT version showed a learning effect toward more advantageous decisions (see **Figure 2** and **Supplementary Table S4**).

The different shapes of the curves have to be discussed against the background of the IGT. Rules for gains and losses of hypothetical money are not told prior to completing the task. Participants must first learn the contingencies of the card decks (i.e. monetary rewards and punishments) *via* trial and error. They then may realize the approximate frequency and magnitude of monetary wins and losses *via* processing the given feedback and, over time, develop a preference for the advantageous card decks arising from the outcomes related to their previous decisions. According to the third hypothesis it was assumed that decision-making would interfere with craving responses toward appetitive food cues and food addiction symptoms. The aforementioned between-group comparisons together with the results of the moderated regression analyses favor this hypothesis. In the incongruent IGT condition, individuals with strong craving responses (i.e. high “urge to eat” toward appetitive food cues, high FCQ-State scores) or a high level of food addiction symptoms (YFAS 2.0) performed worse than individuals with lower craving responses or less symptoms of food addiction.

In conclusion, the findings suggest that food pictures processing interfered with advantageous decision-making in individuals with class 2 or 3 obesity. Particularly those individuals with high craving responses toward appetitive food cues or high levels of food addiction showed less advantageous decision-making in case appetitive food pictures were linked to disadvantageous outcomes. Contrary to our expectations, the present sample showed stronger tendencies to avoid than to approach appetitive food cues. This result is partly in line with the findings of Paslakis et al. (29) who applied an approach-avoidance task (AAT) with high *vs.* low caloric food pictures. In that study, individuals with obesity and binge eating disorder showed an avoidance bias (and no approach bias) for low caloric food cues, while those with obesity only showed an approach bias (and no avoidance bias) for low caloric food. There are some important methodological differences between the study of Paslakis et al. (29) and our study. First, Paslakis et al. (29) used the AAT which is a behavioral reaction time task that assesses both automatic, compatible reactions (avoid negative, approach positive) as well as their regulation in incompatible conditions (approach negative, avoid positive) (53). In the present study, the IGT that measures decision-making under uncertainty and the preference for short-term rewarding choices (30) was administered. Second, Paslakis et al. (29) built two subgroups of people with obesity: with and without binge eating disorder. The subgroup approach was also utilized by others who found impaired decision-making abilities in individuals with obesity and binge eating disorder (20–22). In the present work, we did not focus on binge eating but on the interaction of IGT condition and affective responses to food pictures or food addiction symptoms in predicting decision-making performance. Our findings indicate a negative association between food addiction severity levels and overall scores on the IGT, which resembles the results of Steward et al. (27) who reported about altered decision-making in women with obesity and food addiction.

Based on the current findings it is not possible to determine if the surprising results are typical for bariatric surgery candidates only or for individuals with obesity only. Unfortunately, the study did not include a control group of individuals without obesity, which limits the interpretability. Therefore, a subsequent study was conducted by using the same methodology, but conducted on a sample of participants without obesity.

## Study 2

### Materials and Methods

#### Participants Without Obesity

Inclusion and exclusion criteria were the same as in study 1, except the weight status. Only individuals with normal-weight or pre-obesity, defined as a BMI between 18.49 and 29.99 kg/m^2^, were included. All participants received a compensation of 30€. The addendum to the study protocol regarding the subsequent recruitment and assessment of individuals without obesity was approved by the Institutional Review Board and added in the German Clinical Trials Register.

*A priori* power analysis assuming a large effect size (*d* = 1.00) for between-group differences in IGT net scores based on study 1 indicated that a total sample size of 54 participants is sufficient to reach a 95% power when employing the .05 criterion of statistical significance. Recruitment of participants took place between May 2018 and July 2018 by word-of-mouth and notices in public venues (e.g., hospitals, university).

The initial sample included 62 volunteers who were randomly assigned to the congruent (*n* = 31) or incongruent (*n* = 31) IGT condition. Five participants were dropped from the study due to the following reasons: BMI >30 kg/m^2^ (*n* =1), scoring on the numeric hunger scale >50 (*n* = 3), and technical problems with the IGT (*n* = 1). Data from another three persons were excluded from analyses because these participants had constantly picked cards from a certain IGT card deck that resulted in one empty deck after 60 out of 100 trials. The final group consisted of 54 participants, of whom 28 individuals were randomly assigned to the congruent and 26 to the incongruent IGT condition.

#### Study Procedure

Participants received the same experimental procedure as in study 1, including a cue-reactivity paradigm, the MCST, and the modified IGT (see **Figure 1**).

#### Questionnaires

The same questionnaires as in study 1 were administered, Cronbach’s α in the non-obese sample were: .93 for the FCQ-State (baseline and after completing the IGT), .62 for the YFAS 2.0 (i.e. Kuder-Richardson α for the dichotomous scores of the 11 food addiction symptoms), .82 for the BIS-15, .88 for the EDE-Q, .72 for the PHQ-9, and .84 for the GAD-7.

#### Statistical Analysis

The same statistical analyses were conducted as in study 1.

### Study 2: Results

#### Demographic and Clinical Variables

The sample consisted of 54 volunteers (*n* = 40, 74% women) with a mean age of 39.52 years (*SD* = 12.83, *range* 18–62, *Mdn* = 40.50). Gender (χ^2^ = 0.08, *df* = 1, *p = *.777, φ = 0.02) and age distribution (*t* = 0.91, *df* = 159, *p* = .363, *d* = 0.15) did not differ from the study 1 sample. Most individuals in the present sample reported normal weight (*n* = 34, 63%) and 37% (*n* = 20) had pre-obesity, which was different from study 1 (χ^2^ ≥ 161.00, *df* = 3, *p* <.001, φ = 1.00) due to the inclusion criteria. The following prevalence estimates were found based on questionnaires’ cutoffs: food addiction 3.7% (*n* = 2), eating disorder 16.7% (*n* = 9), major depressive disorder 1.9% (*n* = 1), and anxiety disorder 1.9% (*n* = 1). All prevalence estimates were lower than in study 1 (all χ^2^ ≥ 11.59, *p* ≤.001), while the magnitude of the difference was small to moderate for anxiety disorder (φ = 0.27), moderate for major depressive disorder (φ = 0.42) and food addiction (φ = 0.45), and large for eating disorder (φ = 0.53).

Comparisons between participants in the congruent compared to those in the incongruent IGT condition with respect to demographic and clinical variables did not reveal any significant between-group differences (for details see **Supplementary Table S6**).

#### Subjective Food Pictures Ratings and Food Cravings

Information on food pictures taken from the “Food Pics” image database for experimental research on eating and appetite (36) used in study 2 is provided in **Supplementary Table S1**. The distribution of selected categories of pictures of palatable, processed food or fruit for both IGT conditions is displayed in **Supplementary Figure S2**. Participants in the congruent IGT condition did not differ from those in the incongruent IGT condition with regard to preferred food pictures (χ^2^ = 7.27, df = 6, *p* = .297, φ = .37). Paired-samples *t*-test indicated a lack of significant within-group differences regarding the valence (total sample: *t* = 0.81, *df* = 53, *p* = .422, *d* = 0.13; congruent: *t* = .02, *df* = 27, *p* = .938, *d* <.01; incongruent: *t* = 1.16, *df* = 25, *p* = .255, *d* = .32) and the “urge to eat” (total sample: *t* = 1.07, *df* = 53, *p* = .290, *d* = 0.15; congruent: *t* = .29, *df* = 27, *p* = .774, *d* = .05; incongruent: *t* = 1.80, *df* = 25, *p* = .083, *d* = .34) with respect to the selected pictures showing processed food or fruit compared to images of raw vegetables (for details see **Table 3**). No between-group differences were observed with regard to subjective pictures ratings and craving responses (**Table 3**). MANOVA with the four pictures ratings as dependent variables (valence/urge; palatable, processed food or fruit/raw vegetables) showed no main effect of group (congruent *vs.* incongruent) (Wilk’s λ *F*(4,49) = 0.95, *p* = .660, η² = .05).

**Table 3:** Comparison of hunger, food cravings, food pictures ratings, and general cognitive functions for individuals with normal weight/pre-obesity playing the modified IGT in the congruent *vs.* the incongruent condition (study 2).

	Total sample	IGT condition		*t*	*p*	*d*
		Congruent	Incongruent			
	*N* = 54	*n* = 28	*n* = 26			
	*mean (SD)*	*mean (SD)*	*mean (SD)*			
Hunger^a^	8.37 (11.01)	8.04 (10.29)	8.73 (11.94)	−.23	.819	.06
Appetitive food pictures						
Rating—valence	3.44 (0.65)	3.41 (.70)	3.48 (.62)	−.40	.691	.11
Rating—urge to eat	1.66 (0.67)	1.60 (.62)	1.73 (.72)	−.74	.459	.20
Raw vegetables pictures						
Rating—valence	3.34 (0.79)	3.41 (.89)	3.27 (.69)	.64	.523	.17
Rating—urge to eat	1.56 (0.67)	1.64 (.81)	1.48 (.49)	.85	.398	.23
Food Cravings Questionnaire State						
Baseline^b^	21.20 (8.12)	22.39 (10.09)	19.92 (5.80)	1.09	.280	.30
Post	22.22 (9.15)	22.89 (9.66)	21.50 (8.69)	.56	.581	.15
MCST						
Correct responses	33.85 (6.73)	32.57 (8.05)	35.23 (4.70)	−1.47	.148	.40
Normal errors	6.98 (6.26)	8.11 (7.06)	5.77 (5.13)	1.38	.173	.38
Perseverative errors	4.30 (3.60)	5.11 (4.10)	3.42 (2.79)	1.75	.086	.48

^a^Numeric Hunger Scale, ^b^df = 104, for all comparisons was df = 52.

IGT, Iowa Gambling Task; VAS, Visual Analog Scale; MCST, Modified Card Sorting Test.

In both conditions, no significant changes in food craving as measured with the FCQ-State emerged over the course of the experimental procedure. Repeated measures ANOVA with FCQ-State scores at baseline *vs.* following the experiment as within factor “time” and IGT “condition” (congruent *vs.* incongruent) as between factor were performed. The result indicates no significant “time × condition” effect (Wilk’s λ = 0.99, *F*(1, 52) = 0.42, *p* = .520, η^2^ <.01).

#### Behavioral Tasks Performance

No significant differences between conditions were found regarding general cognitive functions as measured with the MCST (see **Table 3**). With respect to decision-making abilities, individuals in the congruent condition had a significantly lower overall IGT net score (*mean* = −24.43, *SD* = 34.92) than participants in the incongruent condition (*mean* = 18.92, *SD* = 30.56). An independent *t*-test showed that the difference between conditions was statistically significant (*t* = 4.82, *df* = 52, *p* <.001). The effect of the difference was large (*mean difference* = −43.35, 95% CI: −61.40 to −25.30, *d* = 1.34). The overall IGT net scores did not differ significantly between study 1 and study 2 (see **Supplementary Table S7**).

**Figure 4:**
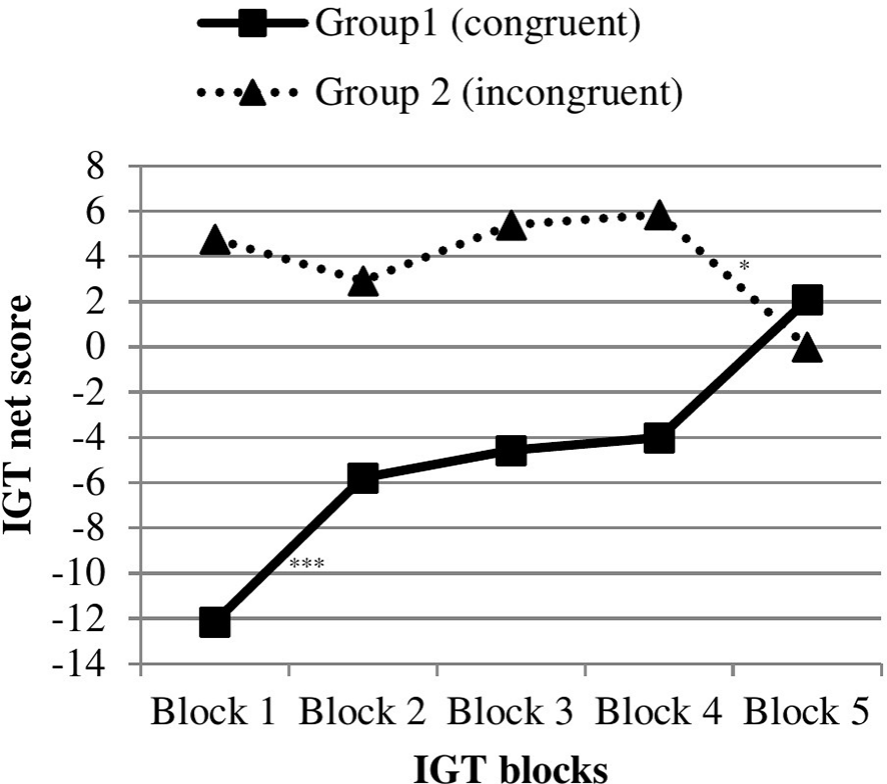
Task performance of individuals with normal weight/pre-obesity across five blocks in the congruent *vs.* incongruent IGT condition (study 2). IGT, Iowa Gambling Task, ***p < .001, *p < .05.

**Figure 4** displays the IGT learning curves. Separate repeated measures ANOVAs for each condition revealed a significant main effect of the within factor “block” in the congruent condition indicating changes in IGT net scores across the five blocks (*F*(4, 24) = 13.35, *p* <.001, η^2^ = .69). Changes in the incongruent condition did not reach statistical significance (*F*(4, 22) = 2.22, *p* = .100), while the magnitude of the effect was large (η^2^ = .29). As illustrated in **Figure 4**, participants in the congruent IGT condition showed a learning effect, but participants in the incongruent condition did not. Repeated measures ANOVA with total IGT net scores of the five blocks as within factor, and IGT condition (congruent *vs.* incongruent) as between factor, revealed a significant block-by-condition effect (Wilk’s λ = 0.65, *F*(4, 49) = 6.47, *p* <.001, η^2^ = .35). Descriptives and test statistics for between-group comparisons (congruent *vs.* incongruent) are reported in **Supplementary Table S3** and for pairwise within–group comparisons in **Supplementary Table S8**.

#### Moderating Variables on Decision-Making Performance

**Supplementary Table S9** shows the bivariate correlations of the overall IGT net scores with other variables. In both IGT conditions, lower IGT net scores were related to higher “urge to eat” toward pictures of palatable, processed food or fruit but not toward images of raw vegetables. Of potential interest is the moderately strong correlation between lower IGT net scores and more symptoms of depression (PHQ-9) in participants in the congruent IGT version compared to those in the incongruent condition. According to the *z*-statistics, however, the correlations did not differ significantly.

Similar to study 1, separate moderated regression analyses were performed with IGT net score as dependent variable, IGT condition as predictor and the following moderators: “urge to eat” (appetitive food pictures rating), food cravings (FCQ-State baseline/post), and symptoms of food addiction (YFAS 2.0). As can be seen in **Table 4**, there were no significant interaction effects. For comparison, the BIS-15, EDE-Q, PHQ-9, or GAD-7 were included as moderators in separate regression models. Again, no significant interaction effects were observed (results not reported here).

**Table 4:** Summary of moderated regression analyses investigating the impact of craving responses, food addiction symptoms, or impulsivity on the relationship between IGT condition (congruent = 1, incongruent = 2) and decision-making outcome (dependent variable: overall IGT net score) in individuals with normal weight/pre-obesity (study 2).

Predictor/Moderator variables	*F*	*R*^2^	*B*	*SE*	β	*t*	*p*
Step 1	23.23^***^	.31					
Condition			43.35	8.99	.56	4.82	<.001
Step 2	19.86^***^	.44					
Condition			46.25	8.23	.59	5.62	<.001
Urge to eat			−21.24	6.21	−.361	−3.421	.001
Step 3	13.02^***^	.44					
Condition			46.27	8.31	.59	5.57	<.001
Urge to eat			−21.50	6.34	−.37	−3.39	.001
Condition × Urge to eat			3.31	12.62	.03	.26	.794
Step 1	23.23^***^	.31					
Condition			43.35	8.99	.56	4.82	<.001
Step 2	12.79^***^	.33					
Condition			41.47	9.02	.53	4.60	<.001
FCQ-baseline			−.76	.55	−.16	−1.39	.170
Step 3	8.37^***^	.33					
Condition			41.58	9.15	.53	4.54	<.001
FCQ-baseline			−.72	.64	−.15	−1.13	.265
Condition × FCQ-baseline			.16	1.30	.02	.12	.903
Step 1	23.23^***^	.31					
Condition			43.35	8.99	.56	4.82	<.001
Step 2	12.89^***^	.34					
Condition			42.36	8.93	.54	4.74	<.001
FCQ-post			−.71	.49	−.16	−1.44	.156
Step 3	9.25^***^	.36					
Condition			42.52	8.88	.54	4.79	<.001
FCQ-post			−.64	.49	−.15	−1.30	.198
Condition × FCQ-post			1.27	.99	.15	1.28	.206
Step 1	23.23^***^	.31					
Condition			43.35	8.99	.56	4.82	<.001
Step 2	11.66^***^	.31					
Condition			43.36	9.05	.56	4.79	<.001
YFAS 2.0			−2.50	4.11	−.07	−.61	.546
Step 3	7.62^***^	.31					
Condition			43.36	9.14	.56	4.74	<.001
YFAS 2.0			−2.52	4.17	−.07	−.60	.549
Condition × YFAS 2.0		R2	.37	8.32	<.01	.04	.965

IGT, Iowa Gambling Task; FCQ, Food Cravings Questionnaire State; YFAS 2.0, Yale Food Addiction Scale 2.0, ^***^p < .001.

### Study 2: Discussion

In this convenience sample with normal weight or pre-obesity, prevalence estimates of food addiction, depressive and anxiety disorders were lower than in population-based samples (48, 49, 54) and lower than in study 1. The proportion of participants showing high EDE-Q scores was relatively high compared to the general population (44), but low compared to individuals with obesity in study 1 or past samples of bariatric surgery candidates (13, 51).

As opposed to study 1, the results revealed no significant differences in terms of subjective appetitiveness ratings of pictures presenting palatable, processed food or fruit compared to pictures showing raw vegetables. This is surprising and needs explanation. High food literacy and a health mindset might have contributed to the lack of differences in valence and “urge to eat” ratings of pictures showing processed food or fruit *vs.* raw vegetables. We will discuss this assumption in more detail blow in the general discussion.

Also in line with study 1, food images processing influenced decision-making under ambiguity. Individuals in the congruent IGT condition performed worse than participants in the incongruent condition, and the magnitude of this difference was large. The overall IGT net scores indicated a strong preference for those card decks that were covered by pictures of raw vegetables and avoidance of card decks with pictures of palatable, processed food or fruit, regardless of IGT condition, i.e. even if the card decks with raw vegetables resulted in more negative outcomes. This finding reflects the strong impact of food pictures on decision-making and resembles the findings of study 1. Again, only within the congruent IGT condition, participants showed a learning effect across the five IGT blocks (see **Figure 4** and **Supplementary Table S8**). They shifted their choices toward advantageous card decks and learned to prefer the good card decks over the bad card decks. This could be interpreted as a decrease of interference between food pictures processing and IGT specific feedback processing (referring to monetary wins and losses) in the congruent condition as the task progressed. As opposed to the results of study 1, no significant interaction effects between IGT condition and craving responses/food addiction symptoms on decision-making performance emerged. Possible reasons for the outcomes presented here are now detailed in the general discussion.

## General Discussion

One of the main findings of the present study is that food pictures processing interfered with advantageous decision-making in individuals with class 2 and 3 obesity. Opposed to our hypothesis, stronger tendencies to avoid than to approach pictures presenting processed, tasty food (= appetitive food cues) were observed, particularly at the beginning of the IGT task. This outcome cannot be considered specific for individuals with obesity given that a similar pattern was found among individuals with normal-weight or pre-obesity (study 2). The overall IGT net scores were comparable between study 1 and study 2, in the congruent as well as the incongruent condition. These outcomes indicate a preference of healthy food cues and not of palatable, processed food cues in both individuals with and without obesity. It is worth considering that the pictures of processed food may have elicited negative expectancies linked to adverse consequences that follow consumption of high-calorie food (e.g., weight, shape, or health concerns). This observation is in line with the approach/avoidance framework of alcohol dependency proposed by Breiner and colleagues (55). According to that model, approach and avoidance are separate dimensions of craving. It is likely that our initial view of craving exclusively in terms of automatic approach tendencies toward appetitive food cues did not capture the complexity of responses activated by food cues, regardless of weight status or potential social desirability effects.

There is also a striking similarity between study 1 and study 2 with respect to changes in IGT net scores across the five blocks with a strong learning curve in the congruent condition and a lack of learning in the incongruent version. In the incongruent condition, the switch from advantageous card decks linked to raw vegetables to disadvantageous decks linked to palatable, processed food or fruit seemed to impede advantageous decision-making. The results were similar between study groups despite the fact that participants in the obesity group admitted on average higher craving responses toward pictures of palatable, processed food or fruit compared to pictures of raw vegetables than the group without obesity, which is in line with the high level of food addiction symptoms in this group and with the literature (14, 15, 17, 19). The interference effect of food pictures processing on advantageous decision-making has been observed particularly in those individuals with class 2 or 3 obesity who exhibited high craving responses toward appetitive food cues or high levels of food addiction. As shown in previous studies, high levels of food addiction may have a negative impact on weight management in this clinical population (56). In the non-obese sample, only two persons met the YFAS 2.0 threshold for food addiction (40, 42). Given the low level of food addiction symptoms and affective responses towards food pictures in that group, it is not surprising that we did not find a moderation effect of craving/food addiction symptoms. Of note, the internal reliability of the YFAS 2.0 (Kuder-Richardson α = .62) was unusually low in study 2 compared to the literature (40, 57).

Study 1 investigated bariatric surgery candidates. According to guidelines, surgery for obesity can be offered to individuals with obesity class 2 or 3 when conventional treatment approaches have failed (58, 59). It can therefore be assumed that participants of study 1 have been suffering from overweight/obesity for many years and have undergone lifestyle modification programs and non-surgical weight loss treatments across their life span (60). Moreover, at the time of the assessment, all participants were in interdisciplinary preoperative outpatient care at one of the cooperating surgical departments. Preoperative care usually includes nutritional counseling and weight management sessions to promote postoperative weight loss or maintenance (61). A plausible explanation for the strong tendency to avoid appetitive food cues is that participants of study 1 certainly have learned that one way to reduce calorie intake is to switch from high-energy food choices to healthier choices.

With respect to study 2, which included persons with normal weight or pre-obesity, the preference for images of raw vegetables (while playing the IGT) could be explained by underlying positive associations with healthy nutrition and avoidance of processed, energy-rich food. In this vein, the present results might be limited by a selection bias. The sample of study 2 may have comprised especially health conscious volunteers with good food literacy. In a choice between processed, tasty food and raw vegetables, choosing the latter may have been more consistent with their lifestyle attitudes and everyday eating habits. Participants’ health mindset could have attenuated attentional bias towards pictures of healthy raw vegetables (62–64). However, such reasoning remains speculative due to the lack of information about these aspects in the present samples. The role of food literacy, food concerns, and health mindset as compared to a palatability mindset should be addressed in future studies.

Another consideration refers to the influence of hunger and satiety. Those participants who scored equal or above 50 on the numeric hunger scale were excluded from the experiment. It might be possible that feelings of hunger would have increased the preference for those card decks that were covered with pictures of palatable, high-calorie food. According to dual-process models of addiction (65, 66), hunger may increase involuntary automatic appetitive processes toward palatable, processed food (bottom-up regulation) while the ability to deliberately suppress automatic pre-potent choices for palatable, processed food (top-down regulation) becomes weaker. The moderating effect of craving responses and food addiction symptoms on decision-making in patients with obesity in the incongruent IGT version (study 1) supports this assumption.

Last but not least, differences in methodology between the present and past studies that made use of a modified IGT version with addiction-related pictures have to be considered. Past studies had used task versions where pornography-, shopping-, or drug-related pictures (34, 35, 52) were compared with neutral control pictures from the IAPS (33). In the present study, the IGT card decks were covered by pictures of palatable, processed food or fruit *vs.* images of raw vegetables. The utilization of neutral IAPS pictures instead of raw vegetables pictures might have led to different results. Given that food is a natural reinforcer (67), one may argue that participants would have more frequently chosen the card decks with food pictures than those with neutral images. However, this was not the focus of the present study that addressed participants’ responses toward palatable, processed, high-energy food compared to healthy food. To design the laboratory task as close as possible to the participants’ food preferences, food stimuli were semi-individualized, which is an advantage of the present work. However, it might be doubtful whether visual food cues can stimulate as much craving responses as olfactory cues or real food, even though meta-analyses indicate that the modality of food cues (e.g., pictures, videos, words, smells) had no moderating effect on craving responses or inhibitory control in past studies (19, 68). This raises the question which study design would best approximate the life circumstances of individuals with obesity. Using a laboratory feeding paradigm before and after performing a decision-making task could be helpful to investigate the assumed interplay between food preferences and general decision-making abilities, taking into account hunger and satiety. The combination of naturalistic studies (e.g., employing ecological momentary assessment of eating behavior) and laboratory decision-making tasks may be another useful approach. Furthermore, the role of potential moderating factors (e.g., acute stress, momentary mood) on food choices should be considered. Loeber et al. (69) found that mood predicted food-associated inhibition deficits in interaction with restrained eating in women with obesity and binge eating using a go/no-go task with food-related *vs.* control stimuli. It is also worth to consider the impact of acute stress on affective responses towards food cues. Similarly to substance use disorders (70, 71), stress-induced changes in information processing may decrease inhibitory control, boost approach tendencies towards specific food (e.g., palatable, junk food) and result in more disadvantageous decisions. When under acute stress, individuals with obesity and addiction-like eating behavior might automatically choose palatable food. The present study was conducted under rest condition. Experimental studies examining the interplay of acute stress (using a standardized stress paradigm), affective responses/craving towards food stimuli, and decision-making could shed light on the complex relationships between those variables. Last but not least, the inclusion of *a priori* defined subgroups (e.g., with *vs.* without binge eating disorder; prior *vs.* following lifestyle intervention/psychotherapy) seems to be appropriate.

Some shortcomings have to be considered when interpreting the results. First, only appetitive food cues were semi-individualized, but non-appetitive cues were not. Second, as mentioned above, a selection bias has to be considered. Third, this work was not conceptualized as a case control study with bariatric surgery candidates and a matched control group of individuals with obesity not seeking surgical treatment and/or a control group without obesity. Study 2 was completed as a subsequent study in view of the surprising results of study 1. Fourth, the relatively high number of invalid IGT data in both studies suggest some difficulties related to the implementation of the modified IGT version or that some participants might have been overstrained or just did not follow the instruction as appropriate. Data from participants with invalid measurements had to be dropped from the study to handle this problem. Moreover, prevalence estimates of food addiction, depressive and anxiety disorders refer to self-ratings.

Taken together, it appears that individuals with and without obesity show a preference for healthy food cues over processed, high-calorie food cues while performing a decision-making task. However, over time, palatable food cues may impede advantageous decision making. The findings indicate that exposure to food cues might interfere with advantageous decision-making, especially in individuals with strong craving responses and high symptoms of food addiction. Further research in the field of obesity should examine how cognitive avoidance tendencies toward processed, high-energy food and approach tendencies toward healthy food can be transferred to real life situations and conventional treatments of obesity.

## Data Availability Statement

The datasets generated for this study are available on request to the corresponding author.

## Ethics Statement

The studies involving human participants were reviewed and approved by the Ethics Committee of the Hannover Medical School. The patients/participants provided their written informed consent to participate in this study.

## Author Contributions

Conceptualization: AM, ML, EW, MZ, SJ-M. Methodology: ML, AM, EW, SM, NL. Assessments: ML, NL, GS, RW. Software: ML, EW, NL, MZ. Data Analysis: AM, EW, SM, ML. Writing—original draft: ML, AM. Writing—review and editing: AM, ML, EW, SM, MZ, SJ-M, GS, RW, NL.

## Funding

This work was supported by the “Else-Kroener-Fresenius Foundation” as part of the structured doctoral program “ClinStrucMed.”

## Conflict of Interest

The authors declare that the research was conducted in the absence of any commercial or financial relationships that could be construed as a potential conflict of interest.
