# Supplementary material for: A Randomized Study of Food Pictures-Influenced Decision-Making Under Ambiguity in Individuals With Morbid Obesity
Source: Front Psychiatry. 2020 Sep 11;11:822. doi: 10.3389/fpsyt.2020.00822 (PMC7518028; doi:10.3389/fpsyt.2020.00822)
Supplement: Supplementary file 1 [file Image_1.pdf]

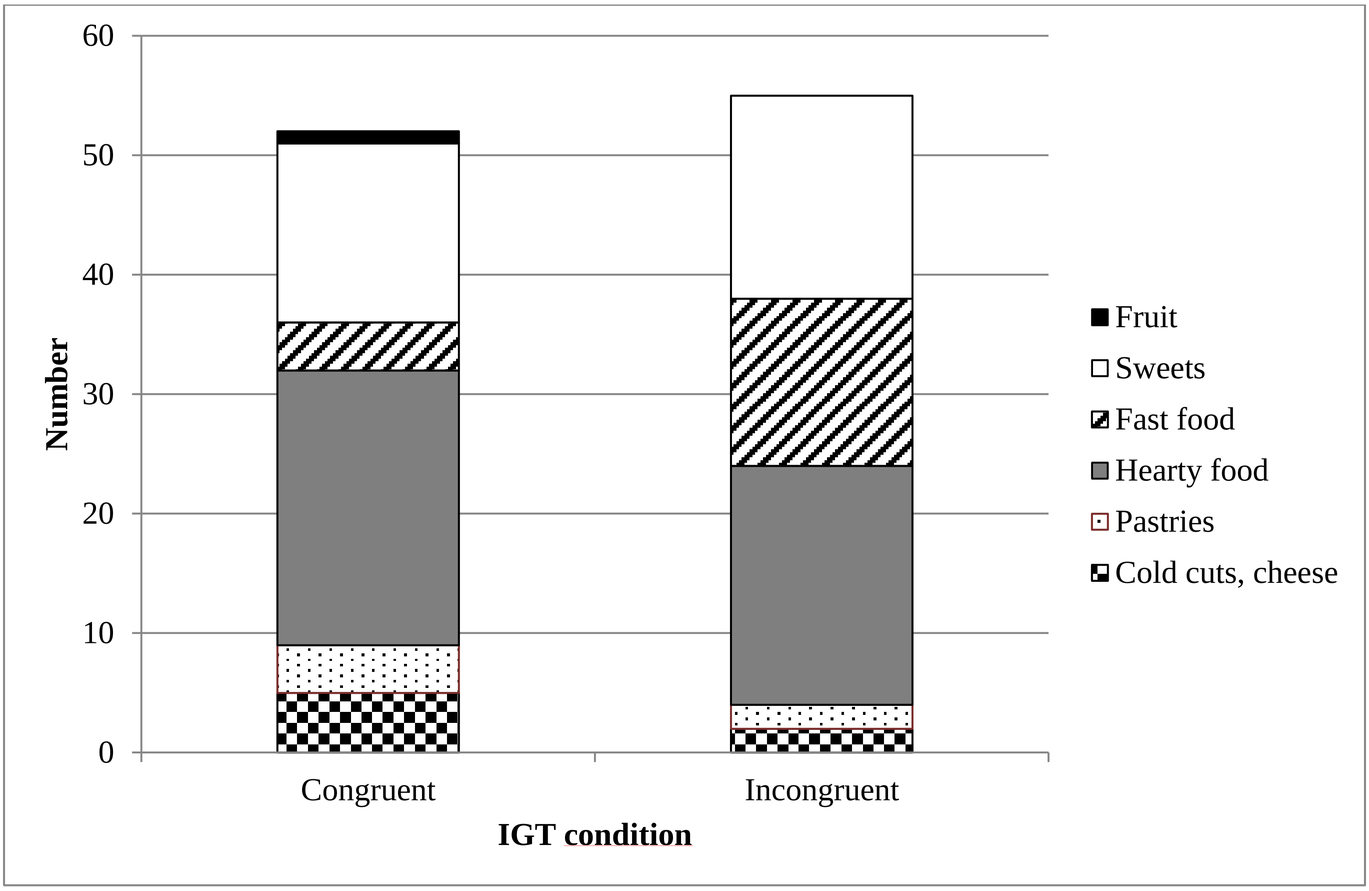

Supplementary Figure S1: Distribution of appetitive food categories selected by individuals with obesity (study 1)

IGT = Iowa Gambling Task

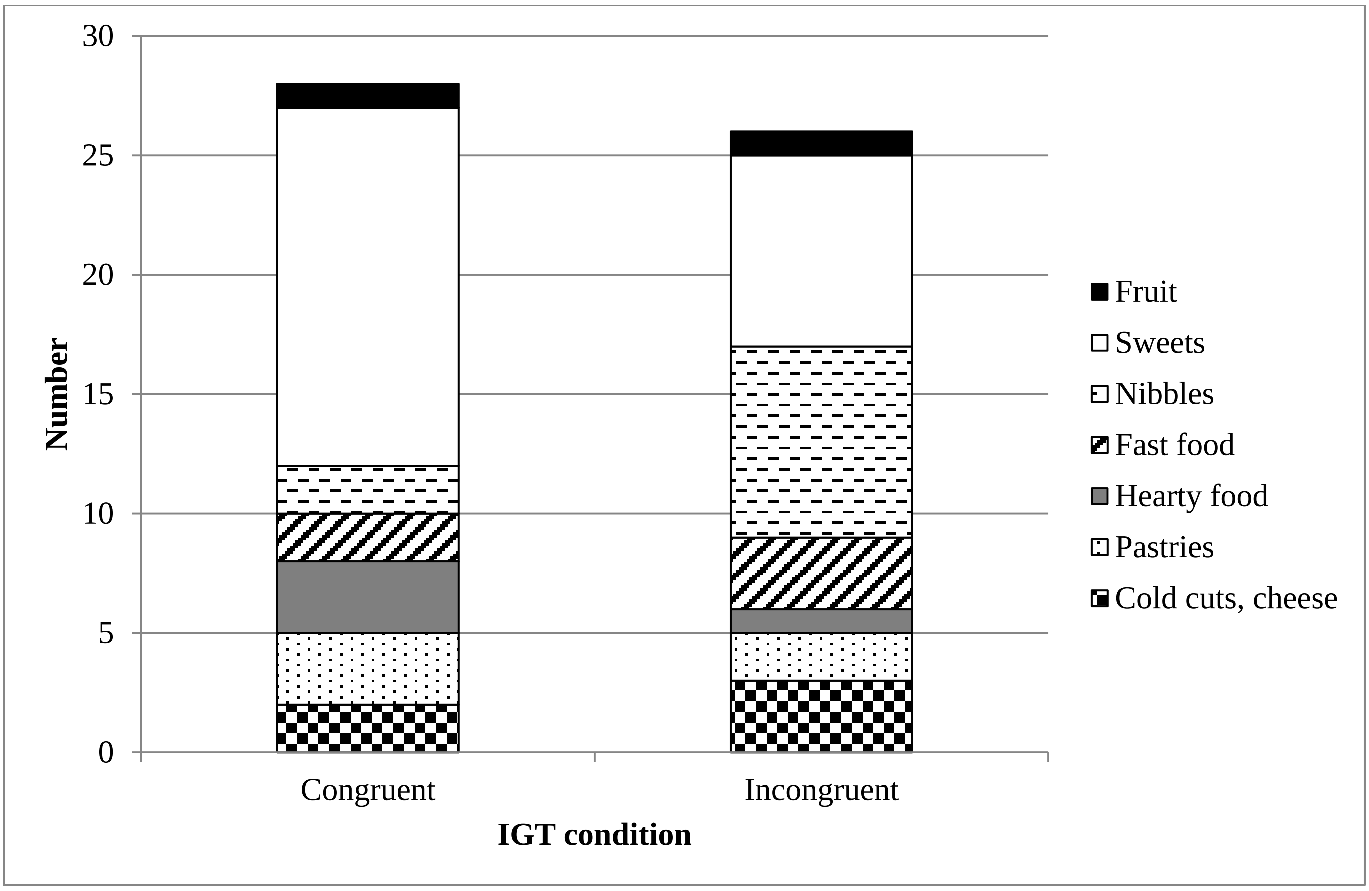

Supplementary Figure S2: Distribution of appetitive food categories selected by individuals with normal weight/pre-obesity (study 2)

IGT = Iowa Gambling Task
