# Supplementary material for: A Randomized Study of Food Pictures-Influenced Decision-Making Under Ambiguity in Individuals With Morbid Obesity
Source: Front Psychiatry. 2020 Sep 11;11:822. doi: 10.3389/fpsyt.2020.00822 (PMC7518028; doi:10.3389/fpsyt.2020.00822)
Supplement: Supplementary file 2 [file Table_1.pdf]

Supplementary Table S1: Food pictures taken from the ‘Food Pics’ image database for experimental research on eating and appetite (Blechert, Meule, Busch, & Ohla, 2014) used in the present study

**Food category**	Fruit	Sweets	Fast Food	Hearty food	Pastries	Cold cuts/ cheese	Salty/nutty nibbles	Raw vegetables
**Food picture numbers from ‘Food Pics’ image database**	0194	0021	0002	0030	0005	0011	0008	0208
	0199	0024	0017	0072	0009	0054	0027	0239
	0204	0025	0022	0143	0013	0064	0043	0242
	0209	0035	0032	0145	0020	0071	0104	0249
	0217	0036	0047	0309	0028	0082	0109	0250
	0221	0083	0052	0311	0040	0098	0110	0251
	0222	0124	0060	0318	0041	0175	0113	0252
	0235	0128	0061	0322	0048	0176	0149	0259
	0245	0147	0065	0337	0074	0329	0150	0263
	0255	0154	0068	0387	0097	0371	0152	0267
	0280	0161	0069	0406	0106	0494	0155	0270
	0281	0286	0081	0484	0135	0498	0180	0367
	0379	0287	0085	0485	0162	0515	0183	0412
	0386	0289	0086	0486	0177	0516	0186	0433
	0396	0291	0087	0493	0189	0517	0416	0438
	0454	0297	0108	0556	0353	0519	0450	0455
	0463	0298	0141	0561	0355	0535	0457	0461
	0479	0336	0188	0562	0422	0536	0494	0477
	0528	0491	0326	0563	0511	0559	0525	0508
	0534	0510	0403	0565	0533	0568	0539	0522

Supplementary Table S2: Comparison of demographic and clinical variables for individuals with obesity playing the IGT in the congruent vs. the incongruent condition (study 1)

		**IGT condition**		***t / χ²* *df* = 105 / 2**	***p***	***d / φ***
		**Congruent** (*n* = 52)	**Incongruent** (*n* = 55)			
Sex, female	*n (%)*	37 (71.2)	40 (72.7)	.03	.856	.02
BMI	*mean (SD)*	46.18 (6.66)	46.72 (6.98)	.41	.684	.08
Class 3 obesity<br>Class 2 obesity	*n (%)*	46 (88.5)<br>6 (11.5)	46 (83.6)<br>9 (16.4)	.52	.472	.07
Age	*mean (SD)*	42.19 (11.78)	40.56 (11.57)	.72	.472	.14
More than 11 school years	*n (%)*	17 (32.7)	15 (27.3)	.76	.685	.08
Partnership status, single	*n (%)*	16 (30.8)	15 (27.3)	.16	.690	.04
Native language, German	*n (%)*	47 (90.4)	47 (85.5)	.61	.435	.07
BIS-15	*mean (SD)*	30.40 (6.49)	32.89 (7.19)	1.87	.064	.36
YFAS 2.0	*mean (SD)*	4.06 (3.25)	3.65 (3.08)	.66	.512	.13
Probable food addiction	*n (%)*	29 (55.8)	23 (41.8)	2.08	.149	.14
EDE-Q	*mean (SD)*	3.11 (0.94)	2.98 (1.07)	.66	.508	.13
Probable eating disorder	*n (%)*	39 (75.0)	39 (70.9)	.23	.634	.05
PHQ-9	*mean (SD)*	9.86 (5.62)	8.47 (5.80)	1.26	.210	.24
Major depressive disorder	*n (%)*	25 (48.1)	20 (36.4)	1.50	.220	.12
GAD-7	*mean (SD)*	6.19 (5.25)	6.14 (5.39)	.05	.964	.01
General anxiety disorder	*n (%)*	13 (25.0)	11 (20.0)	.38	.535	.06

Note. IGT = Iowa Gambling Task, BIS-15 = Barratt Impulsiveness Scale, YFAS 2.0 = Yale Food Addiction Scale 2.0, EDE-Q = Eating Disorder Examination-Questionnaire, PHQ-9 = 9-item depression Patient Health Questionnaire, GAD-7 = 7-item Generalized Anxiety Disorder Scale

Supplementary Table S3: Between-group comparison of task performance across 5 blocks in the congruent vs. incongruent IGT condition for study 1 and study 2

	IGT condition		Between-group comparison congruent vs. incongruent IGT condition		
	**Congruent**	**Incongruent**			
Study 1: Patients with obesity	*n* = 52	*n* = 55			
	mean (SD)	*mean (SD)*	$F_{(1)}$	*p*	η^2
Block 1	-9.62 (9.26)	8.15 (11.29)	78.60	< .001	.43
Block 2	-4.08 (9.32)	5.89 (9.86)	28.80	< .001	.21
Block 3	-5.50 (8.84)	5.42 (10.52)	33.60	< .001	.24
Block 4	-3.54 (8.95)	7.27 (9.46)	36.78	< .001	.26
Block 5	1.69 (8.26)	0.76 (11.22)	0.23	.629	< .01
Study 2: Patients without obesity	*n* = 28	*n* = 26			
	mean (SD)	*mean (SD)*	$F_{(1)}$	*p*	η^2
Block 1	-12.14 (7.42)	4.77 (11.41)	42.27	< .001	.45
Block 2	-5.79 (10.16)	2.92 (9.26)	10.78	.002	.17
Block 3	-4.57 (11.85)	5.38 (8.78)	12.15	.001	.19
Block 4	-4.00 (12.58)	5.85 (8.60)	11.11	.002	.18
Block 5	2.07 (10.60)	00 (11.10)	.49	.486	< .01

Supplementary Table S4: Pairwise within-group comparison of IGT net scores across 5 blocks in the congruent vs. incongruent IGT condition for patients with obesity (study 1)

Congruent condition							Incongruent condition						
IGT block		Mean difference	*SE*	*p*	95% CI		IGT block		Mean difference	*SE*	*p*	95% CI	
**1**	**2**	-5.54	1.38	< .000	-8.30	-2.77	**1**	**2**	2.25	.97	.023	.32	4.19
	**3**	-4.11	1.32	.003	-6.78	-1.45		**3**	2.73	1.18	.025	.36	5.10
	**4**	-6.08	1.56	< .000	-9.21	-2.94		**4**	.87	1.28	.497	-1.69	3.43
	**5**	-11.31	1.79	< .000	-14.91	-7.71		**5**	7.38	2.42	.004	2.54	12.23
**2**	**1**	5.54	1.38	< .000	2.77	8.30	**2**	**1**	-2.25	.97	.023	-4.19	-.32
	**3**	1.42	1.12	.209	-.82	3.67		**3**	.47	.97	.628	-1.47	2.42
	**4**	-.54	1.34	.689	-3.23	2.15		**4**	-1.38	1.00	.173	-3.39	.62
	**5**	-5.77	1.85	.003	-9.49	-2.05		**5**	5.13	2.36	.034	.39	9.87
**3**	**1**	4.11	1.32	.003	1.45	6.78	**3**	**1**	-2.73	1.18	.025	-5.10	-.36
	**2**	-1.42	1.12	.209	-3.67	.82		**2**	-.47	.97	.628	-2.42	1.47
	**4**	-1.96	.94	.042	-3.85	-.07		**4**	-1.85	.83	.030	-3.52	-.18
	**5**	-7.19	1.77	< .000	-10.74	-3.64		**5**	4.65	2.22	.041	.20	9.11
**4**	**1**	6.08	1.56	< .000	2.94	9.21	**4**	**1**	-.87	1.28	.497	-3.43	1.69
	**2**	.54	1.34	.689	-2.15	3.23		**2**	1.38	1.00	.173	-.62	3.39
	**3**	1.96	.94	.042	.07	3.85		**3**	1.85	.833	.030	.18	3.52
	**5**	-5.23	1.66	.003	-8.55	-1.91		**5**	6.51	2.18	.004	2.14	10.88
**5**	**1**	11.31	1.79	< .000	7.71	14.91	**5**	**1**	-7.38	2.42	.004	-12.23	-2.54
	**2**	5.77	1.85	.003	2.05	9.49		**2**	-5.13	2.36	.034	-9.87	-.39
	**3**	7.19	1.77	< .000	3.64	10.74		**3**	-4.65	2.22	.041	-9.11	-.20
	**4**	5.23	1.66	.003	1.91	8.55		**4**	-6.51	2.18	.004	-10.88	-2.14

Supplementary Table S5: Bivariate correlations of overall IGT net scores with hunger, food pictures ratings, food cravings and clinical variables for individuals with obesity playing the modified IGT in the congruent vs. the incongruent condition (study 1)

	**IGT condition**			
	**Congruent** (*n* = 52)	**Incongruent** (*n* = 55)	***z***	***p***
Hunger[a]	-.05	-.21	0.82	.206
Appetitive food pictures				
Rating - valence	.12	-.31*	2.22	.013
Rating – urge to eat	.01	-.46**	2.55	.005
Raw vegetables pictures				
Rating –valence	-.15	.35**	-2.59	.005
Rating – urge to eat	-.01	-.03	0.10	.460
FCQ-State				
Baseline	.03[b]	-.42**	2.34	.008
Post	.16	-.51**	3.64	< .001
YFAS 2.0	-.01	-.40**	2.08	.019
BIS-15	.00	-.24	1.23	.109
EDE-Q	-.37**	-.24	-0.72	.235
PHQ-9	-.19	-.36**	0.93	.177
GAD-7	-.14	-.39**	0.93	.177

Note. [a] Numeric Hunger Scale, [b] *n* = 51
IGT = Iowa Gambling Task, FCQ = Food Cravings Questionnaire, YFAS 2.0 = Yale Food Addiction Scale 2.0, BIS-15 = Barratt Impulsiveness Scale, EDE-Q = Eating Disorder Examination-Questionnaire, PHQ-9 = 9-item depression Patient Health Questionnaire, GAD-7 = 7-item Generalized Anxiety Disorder Scale
*$p \leq .05$; **$p \leq .01$

Supplementary Table S6: Comparison of demographic and clinical variables for individuals with normal weight/pre-obesity playing the modified IGT in the congruent vs. the incongruent condition (study 2)

		**IGT condition**		***t* / χ^2 *df* = 52 / 2**	***p***	***d* / *φ***
		**Congruent** (*n* = 28)	**Incongruent** (*n* = 26)			
Sex, female	*n (%)*	20 (71.4)	20 (76.9)	.21	.645	.06
BMI	*mean (SD)*	23.85 (2.74)	23.84 (2.75)	.01	.989	
Normal weight<br>Pre-obesity	*n (%)*	18 (64.3)<br>10 (35.7)	16 (61.5)<br>10 (38.5)	.04	.835	.03
Age	*mean (SD)*	40.64 (13.33)	38.31 (12.40)	.66	.509	< .01
More than 11 school years	*n (%)*	22 (78.6)	22 (84.6)	.33	.568	.08
Partnership status, single	*n (%)*	11 (39.3)	6 (23.1)	1.64	.200	.17
Native language German	*n (%)*	25 (89.3)	25 (96.2)	.93	.336	.13
BIS-15	*mean (SD)*	28.29 (6.52)	29.27 (5.39)	.60	.550	.16
YFAS 2.0	*mean (SD)*	.57 (1.07)	.58 (1.17)	.02	.986	< .01
Probable food addiction	*n (%)*	1 (3.6)	1 (3.8)	< .01	.957	< .01
EDE-Q	*mean (SD)*	1.46 (0.94)	1.08 (0.98)	1.47	.146	.40
Probable eating disorder	*n (%)*	4 (14.3)	5 (19.2)	.24	.626	.07
PHQ-9	*mean (SD)*	2.82 (2.62)	2.58 (2.25)	.37	.716	.10
Major depressive disorder	*n (%)*	1 (3.6)	0 (0.0)			
GAD-7	*mean (SD)*	3.18 (3.61)	2.38 (2.28)	.96	.343	.26
General anxiety disorder	*n (%)*	1 (3.6)	0 (0.0)			

Supplementary Table S7: Comparison of IGT net scores for individuals with obesity (study 1) and individuals with normal weight/pre-obesity (study 2)

IGT condition	Study 1	Study 2	Comparison study 1 vs. study 2		
	mean (SD)	*mean (SD)*		*p*	*d*
Congruent	-21.04 (28.69)	-24.43 (34.92)	$t_{(78)} = 0.47$	.642	0.11
Incongruent	27.49 (35.08)	18.92 (30.86)	$t_{(79)} = 1.06$	.290	0.25

Supplementary Table S8: Pairwise within-group comparison of IGT net scores across 5 blocks in the congruent vs. incongruent IGT condition for individuals with normal weight/pre-obesity (study 2)

Congruent condition							Incongruent condition						
IGT block		*mean difference*	*SE*	*p*	95% CI		IGT block		*mean difference*	*SE*	*p*	95% CI	
**1**	**2**	-6.36	1.44	< .000	-9.30	-3.41	**1**	**2**	1.85	2.13	.395	-2.55	6.24
	**3**	-7.57	2.10	.001	-11.88	-3.26		**3**	-.61	2.15	.777	-5.05	3.82
	**4**	-8.14	2.32	.002	-12.90	-3.39		**4**	-1.08	1.98	.591	-5.15	2.99
	**5**	-14.21	2.58	< .000	-19.52	-8.91		**5**	4.77	3.35	.167	-2.13	11.67
**2**	**1**	6.36	1.44	< .000	3.41	9.30	**2**	**1**	-1.85	2.13	.395	-6.24	2.55
	**3**	-1.21	1.99	.548	-5.31	2.88		**3**	-2.46	1.24	.058	-5.01	.09
	**4**	-1.79	2.12	.408	-6.14	2.57		**4**	-2.92	1.78	.112	-6.58	.73
	**5**	-7.86	3.10	.017	-14.21	-1.50		**5**	2.92	3.32	.388	-3.92	9.77
**3**	**1**	7.57	2.10	.001	3.26	11.88	**3**	**1**	.61	2.15	.777	-3.82	5.05
	**2**	1.21	1.99	.548	-2.88	5.31		**2**	2.46	1.24	.058	-.09	5.01
	**4**	-.57	1.12	.615	-2.87	1.73		**4**	-.46	1.28	.721	-3.09	2.17
	**5**	-6.64	3.22	.049	-13.25	-.03		**5**	5.38	2.94	.079	-.67	11.44
**4**	**1**	8.14	2.32	.002	3.39	12.90	**4**	**1**	1.08	1.98	.591	-2.99	5.15
	**2**	1.79	2.12	.408	-2.57	6.14		**2**	2.92	1.78	.112	-.73	6.58
	**3**	.57	1.12	.615	-1.73	2.87		**3**	.46	1.28	.721	-2.17	3.09
	**5**	-6.07	3.14	.064	-12.52	.37		**5**	5.85	2.71	.041	.27	11.42
**5**	**1**	14.21	2.58	< .000	8.91	19.52	**5**	**1**	-4.77	3.35	.167	-11.67	2.13
	**2**	7.86	3.10	.017	1.50	14.21		**2**	-2.92	3.32	.388	-9.77	3.92
	**3**	6.64	3.22	.049	.03	13.25		**3**	-5.38	2.94	.079	-11.44	.67
	**4**	6.07	3.14	.064	-.37	12.52		**4**	-5.85	2.71	.041	-11.42	-.27

Supplementary Table S9: Bivariate correlations of overall IGT netscores with hunger, food pictures ratings, food cravings and clinical variables for individuals with normal weight/pre-obesity playing the modified IGT in the congruent vs. the incongruent condition (study 2)

	**IGT condition**			
	**Congruent** (*n* = 28)	**Incongruent** (*n* = 26)	***z***	***p***
Hunger[a]	-.03	.04	0.24	.404
Appetitive food pictures				
Rating - valence	-.17	-.23	0.22	.414
Rating – urge to eat	-.41*	-.46*	0.22	.415
Raw vegetables pictures				
Rating –valence	-.24	.08	-1.125	.130
Rating – urge to eat	-.29	-.06	-0.82	.205
Food Cravings Questionnaire State				
Baseline	-.23	-.12	-0.39	.347
Post	-.35	< .01	-1.26	.103
YFAS 2.0	-.08	-.09	0.03	.486
BIS-15	-.29	-.38	0.35	.363
EDE-Q	-.01	-.26	0.89	.188
PHQ-9	-.48**	-.31	-0.70	.242
GAD-7	-.19	-.26	0.25	.399

Note. [a] Numeric Hunger Scale
IGT = Iowa Gambling Task, FCQ = Food Cravings Questionnaire, YFAS 2.0 = Yale Food Addiction Scale 2.0, BIS-15 = Barratt Impulsiveness Scale, EDE-Q = Eating Disorder Examination-Questionnaire, PHQ-9 = 9-item depression Patient Health Questionnaire, GAD-7 = 7-item Generalized Anxiety Disorder Scale
*$p \leq .05$; **$p \leq .01$
